# Launching *BJR*|*Artificial intelligence*: an editorial

**DOI:** 10.1093/bjrai/ubae002

**Published:** 2024-03-04

**Authors:** Heang-Ping Chan, Issam El Naqa

**Affiliations:** Department of Radiology, The University of Michigan, Ann Arbor, MI 48109, United States; Moffitt Cancer Center, Tampa, FL 33612, United States

The past few years have witnessed extraordinary growth in artificial intelligence (AI) technologies such as machine learning and natural language processing, and their application across the board with profound impact on many diverse sectors including healthcare and radiological sciences.^1^ AI is a transformative technology that promises to improve the wellbeing of humankind by automating mundane tasks, improving decision-making, and driving digital innovation. However, AI also carries many salient risks such as privacy violation and algorithmic bias that may negatively impact society.^2^ The rigor of the underlying scientific developments holds the keys to unlock AI’s potential and provide safeguards for its safe implementation in healthcare, and in radiological sciences in particular.^3^ The latter has been on the forefront of AI developments and implementations for decades and is currently reaping the rejuvenation of the new generation of AI technologies.^4^^,^^5^

Building on its remarkable heritage in radiology, the British Institute of Radiology have launched a new open access journal, *BJR*|*Artificial Intelligence*, as a complementary effort to its flagship publication (*BJR*) and sister journals (*BJR*|*Open* and *BJR*|*Case Reports*) to cover the role of AI in radiological sciences. As co-founding Editors-in-Chief, we would like to welcome you to the inaugural volume and issue of this exciting new journal.

*BJR*|*Artificial Intelligence* aims to target a broad multidisciplinary audience who is interested in up-to-date information and forward-looking technical innovations and clinical applications of artificial intelligence in medical imaging, radiotherapy, radiation oncology, medical physics, radiobiology, radiogenomics, and related sciences, as well as important issues in ethics, transparency, fairness, and responsible use of AI in healthcare that will impact the trustworthiness and adoption of AI in the clinic. In order to support the rigorous scientific mission of the journal, its Editorial Board members are comprised of international leaders in the areas of diagnostic radiology, radiotherapy and oncology, medical physics, machine learning/AI, image processing, digital pathology and informatics.

The rigorous peer review process follows the same double-anonymized process undertaken by all BIR journals to ensure clear and unbiased description of methods and materials and thorough reporting of analysis and validated results. The journal is open access, where published articles are made freely available to read, reuse and share online, which will allow for higher visibility and improved impact. The journal supports the publication of high-quality original Research Articles, Reviews, and Systematic Reviews, as well as Technical Implementation articles, Data Resources for AI, Guidelines and Recommendations, Practice and Policy, and Opinion articles.

It is our vision as Editors-in-Chief that *BJR*|*Artificial Intelligence* will serve as a hub for innovative and original AI work in radiological sciences and provide reliable references on its successful and safe implementation in clinical practice, thereby playing a pivotal role in advancing AI in healthcare and benefiting both patients and healthcare professionals. We look forward to seeing the journal grow and evolve over the coming years and to providing a meaningful and impactful contribution to AI research within radiology and radiation oncology.

## References

[1] Topol EJ. High-performance medicine: the convergence of human and artificial intelligence. Nat Med. 2019;25(1):44-56. doi:10.1038/s41591-018-0300-7 30617339

[2] Drabiak K , Kyzer S, Nemov V, El Naqa I. AI and machine learning ethics, law, diversity, and global impact. Br J Radiol. 2023;96(1150):20220934. doi:10.1259/bjr.20220934 37191072 PMC10546451

[3] El Naqa I , Karolak A, Luo Y, et al Translation of AI into oncology clinical practice. Oncogene. 2023;42(42):3089-3097. doi:10.1038/s41388-023-02826-z 37684407 PMC12516697

[4] El Naqa I , Drukker K. AI in imaging and therapy: innovations, ethics, and impact—introductory editorial. Br J Radiol. 2023;96(1150):20239004. doi:10.1259/bjr.20239004 38011226 PMC10546442

[5] Chan HP , Samala RK, Hadjiiski LM, Zhou C. Deep learning in medical image analysis. In: Lee G., Fujita H., eds. Deep Learning in Medical Image Analysis. Advances in Experimental Medicine and Biology. Vol 1213. Springer, Cham; 2020:3–21. 10.1007/978-3-030-33128-3_1 PMC7442218 32030660

